# A Review of Physical Layer Security Techniques for Internet of Things: Challenges and Solutions

**DOI:** 10.3390/e20100730

**Published:** 2018-09-23

**Authors:** Li Sun, Qinghe Du

**Affiliations:** 1 School of Electronic and Information Engineering, Xi’an Jiaotong University, Xi’an 710049, China; 2 State Key Laboratory of Integrated Services Networks, Xidian University, Xi’an 710071, China; 3 National Simulation Education Center for Communications and Information Systems, Xi’an Jiaotong University, Xi’an 710049, China

**Keywords:** Internet of Things, physical layer security, anti-eavesdropping, noise aggregation, constellation rotation, fountain code

## Abstract

With the uninterrupted revolution of communications technologies and the great-leap-forward development of emerging applications, the ubiquitous deployment of Internet of Things (IoT) is imperative to accommodate constantly growing user demands and market scales. Communication security is critically important for the operations of IoT. Among the communication security provisioning techniques, physical layer security (PLS), which can provide unbreakable, provable, and quantifiable secrecy from an information-theoretical point of view, has drawn considerable attention from both the academia and the industries. However, the unique features of IoT, such as low-cost, wide-range coverage, massive connection, and diversified services, impose great challenges for the PLS protocol design in IoT. In this article, we present a comprehensive review of the PLS techniques toward IoT applications. The basic principle of PLS is first briefly introduced, followed by the survey of the existing PLS techniques. Afterwards, the characteristics of IoT are identified, based on which the challenges faced by PLS protocol design are summarized. Then, three newly-proposed PLS solutions are highlighted, which match the features of IoT well and are expected to be applied in the near future. Finally, we conclude the paper and point out some further research directions.

## 1. Introduction

The Internet of Things (IoT) was originally proposed in 1999 as a means to realize inter-connection and information exchange among devices. Nowadays, it acts as a key enabler for smart cities, intelligent transportation systems, precision medicine, smart grids, etc. [1,2]. Moreover, the emergence of the fifth generation (5G) mobile communications techniques [3,4,5] with improved data rate and delay performance will usher in the “Ubiquitous IoT Era”, creating diverse new IoT applications, markets, and business models [6].

The massive deployment of IoT makes information security unprecedentedly important [7]. Network security techniques are crucial not only for maintaining the regular operations of the networks but also for realizing secure delivery of the services over the networks. Moreover, the servitization of information security will make security a new type of service like voice and data. For example, users can “buy” the security level based on their requirements and the cost. This will provide a more comprehensive quality-of-service (QoS) guarantee for heterogenous users, making information security a profit point for mobile operators. Traditional network security solutions rely heavily on the cryptographic technologies at higher layers of the protocol stack. Although the cryptographic approach is a popular method that is widely used for wired networks (e.g., computer networks) and infrastructure-based wireless networks (e.g., cellular networks), it is not fully suited to future IoT, which will be elaborated on in the following.

First, IoT is composed of a large number of low-cost devices. The IoT devices are typically equipped with limited storage memory and powered with batteries, which in turn yield very limited capabilities in terms of computing and communications. As a result, complicated cryptographic protocols and sophisticated encryption/decryption algorithms are prohibited from being used. Second, IoT is a large-scale network supporting massive connections. As indicated by 3GPP TR 45.820 Technical Specification [8], future IoT applications need to accommodate millions of IoT devices within a cellular cell. Moreover, although the processing capabilities as well as the communications range of individual IoT devices (e.g., sensors) are rather limited, the network as a whole should satisfy the wide-range coverage requirement such that the local sensed data can be delivered to the remote control center for further processing. To meet this demand, the network transmission protocols have to incorporate many new features such as multi-hop routing, cooperative relaying, dynamic access, etc. This makes the IoT highly heterogenous and dynamic. In such a network setting, it is extremely difficult to manage and distribute the secret keys. Third, due to the various application scenarios ranging from environmental monitoring and industrial control to telemedicine and inter-vehicular communications, IoT is expected to support diversified services. Different services have totally different requirements of QoS and security levels. For example, online payment calls for a much higher security level than the ordinary web browsing service does. However, encryption-based methods only provide “binary” security levels. The transmitted information is perfectly secured if the key can be securely exchanged and fully intercepted otherwise. Thus, service-oriented and user-centric security guarantee can not be achieved.

Unlike the traditional cryptographic approaches, physical layer security (PLS) takes advantage of the intrinsic characteristics of wireless channels, such as noise, fading, and interference, to boost the signal reception at the legitimate receiver and degrade the received signal quality at the eavesdropper, and realizes the keyless secure transmission via signal design and signal processing [9,10]. Compared with the cryptographic approaches, PLS techniques have the following advantages. First, PLS schemes do not rely on the encryption/decription operations, thus overcoming the difficulty in the distribution and management of secret keys in large-scale heterogeneous IoT. Second, PLS techniques can fully exploit the characteristics of wireless channels to realize adaptive signal design and resource allocation, thus providing flexible security-level configurations and QoS guarantee. Third, PLS techniques only need to complete the relatively simple signal processing algorithms, which incurs less overhead compared to the encryption-based method does. Based on the above discussions, it can be found that PLS is a promising solution to secure the future IoT.

Although the research of PLS techniques has reached fruitful outcomes, it is still challenging to develop the PLS solutions for IoT applications. In particular, IoT has four unique features: low-cost, wide-range coverage, massive connections, and diversified services. How to design PLS strategies that well match these four features remains an open problem. In this article, we would like to present a comprehensive review of the physical layer security techniques for Internet of Things. The primary goal of this article is to provide an avenue for people trying to educate themselves in the state of the art in this area. The scope of this article mainly includes the introduction of the basic principle of physical layer security, the representative PLS techniques, the unique challenges faced by PLS protocol design, and the emerging IoT-oriented PLS solutions. The remainder of this article is organized as follows. In Section 2, the information-theoretical fundamentals of physical layer security is briefly introduced, followed by the literature survey of major achievements in PLS research. In Section 3, the challenges of PLS protocol design for IoT are discussed. Section 4 highlights some emerging PLS techniques that are well suited to the unique features of IoT. Finally, we conclude the paper and point out several further directions in IoT-oriented PLS research in Section 5.

## 2. Literature Review of PLS Techniques for IoT

### 2.1. Information-Theoretical Fundamentals of Physical Layer Security

The research of physical layer security can be traced back to the secret communications theory established by Shannon [11]. According to Shannon’s theory, the system is considered to be in perfect secrecy if the following condition is satisfied:

(1) H(M|X)=H(M),

where H(M) and H(M|X) are the entropy of the message *M* and the conditional entropy of *M* conditioned on eavesdropper’s observation *X*, respectively. To achieve perfect secrecy, the code that is used to encode the message must be independent of the message itself. One feasible approach to realize this is the "one-time pad" encryption, where each secret-key bit is XORed with each message bit to produce the transmitted codeword *X*. This requirement is too demanding in many applications. Unlike Shannon’s work, Aaron Wyner proposed a noisy wiretap channel model in 1975 [12]. The noisy wiretap channel is composed of one legitimate transmitter (Alice), one legitimate receiver (Bob), and one eavesdropper (Eve). For this model, Wyner formulated the condition for secure transmission, i.e., the transmission is information-theoretically secure if the decoding error probability at the legitimate receiver can be arbitrarily small while no source information can be obtained by the eavesdropper. The maximum rate under which the above condition can be met is termed as the secrecy capacity, which characterizes the performance limits for secure transmissions in noisy channels. In Gaussian wiretap channels, the secrecy capacity $C_{s}$ equals the difference in the Shannon capacities between the legitimate (Alice–Bob) link and the eavesdropping (Alice–Eve) link, i.e.,

(2) $$C_{s}={\left[\log\left(1+{\mathrm{SNR}}_{B}\right)-\log\left(1+{\mathrm{SNR}}_{E}\right)\right]}^{+},$$

where ${\left[x\right]}^{+}=\max\left(x,0\right)$, and ${\mathrm{SNR}}_{B}$ and ${\mathrm{SNR}}_{E}$ are the received signal-to-noise ratios (SNRs) at Bob and Eve, respectively. Based on Label (2), the keyless secure communications can be realized as long as the channel quality of the legitimate link is advantageous over that of the eavesdropping link. Therefore, Ref. [12] laid the foundation for the PLS technique research.

However, throughout the long period since the publication of Wyner’s work, the PLS technique has not attracted much attention. This is attributed to the following reasons: first, the achievability of the secrecy capacity relies on the use of stochastic coding. However, it is extremely difficult to construct practical stochastic codes with affordable complexity. Second, in order to achieve the positive secrecy capacity, the received SNR at the legitimate user must be strictly higher than that at the eavesdropper, which is hard to be guaranteed in wireless environments. Last but not the least, shortly after the concept of secrecy capacity was proposed, Diffie and Hellman devised the public-key cryptography, which relies on mathematical functions believed hard to compute and has dominated security research since its appearance. Due to the above reasons, the research of information-theoretical security touched a low point in the 1970s–1980s. During that period, the representative outcomes in the area of information-theoretic security mainly include [13,14]. To be specific, Ref. [13] analyzed the secrecy capacity for the physically degraded Gaussian wiretap channel, and Ref. [14] generalized Wyner’s results to broadcast channels.

Since the 1990s, with the widespread applications of wireless communications techniques and the increasing popularity of wireless services, the security issue of wireless networks is becoming more and more important, which reignites the research interests in information-theoretical security. In this context, significant advances have been achieved in information-theoretical security studies, mainly including the secrecy capacity analysis for various network models. Ref. [15] established the secrecy capacity theory for Gaussian broadcast channels; Refs. [16,17] analyzed the secrecy capacity for multi-antenna channels; Ref. [18] discussed the secrecy capacity over quasi-static fading channels; Ref. [19] investigated the secrecy capacity over block fading channels without the knowledge of the eavesdropper channel; Ref. [20] derived the secrecy capacity over frequency selective channels; Ref. [21] analyzed the impact of noisy channel feedback on the eavesdropper; Ref. [22] discussed the secure transmission problems with limited feedback.

With the development of multi-user information theory, the focus of information-theoretic security research is also shifted from the point-to-point systems to multi-user systems. In multi-user systems, the inter-user cooperation and interference exist among different users, which brings in great challenges to the analysis of the system secrecy capacity and the design of PLS strategies, and provides important opportunities as well. Ref. [23] proposed the notion of cooperative jamming, and analyzed the achievable secrecy performance for two-way Gaussian wiretap channel and Gaussian multiple-access (MAC) channel with multiple eavesdroppers. Ref. [24] studied the trade-off between cooperation and secrecy in relay channels with secret messages. Ref. [25] analyzed the secrecy rate of cognitive channels with security constraints, where the capacity-equivocation regions were obtained for the discrete memoryless cognitive interference channel and the Gaussian cognitive interference channel.

The aforementioned research outcomes in information-theoretic studies lay the foundation for the design of practical wiretap coding strategies and anti-eavesdropping transmission schemes, and provide the analytical tools for the performance evaluation of the physical layer security techniques.

### 2.2. PLS Schemes for IoT: State of the Art

So far, plenty of PLS schemes have been developed in literature, mainly including the artificial noise injection, the secure beamforming/precoding, the anti-eavesdropping signal design, the cooperation-based secure transmission techniques, power allocation and resource allocation schemes, etc. In this subsection, we would like to present a literature review of the PLS schemes that are applicable to IoT. For readers that are interested in more details about the existing PLS techniques, please refer to [9,26] and references therein.

#### 2.2.1. Artificial Noise Injection

The principle of the artificial noise (AN) injection approach is to simultaneously send the information-bearing signal and the AN to degrade the performance of the eavesdropper. The information-bearing signal and the AN are injected into the range space and the null space of the legitimate user’s channel matrix, respectively. In this manner, the AN only deteriorates the eavesdropper but has little detrimental impact on the legitimate receiver [27]. AN injection is an effective means to create the channel quality advantage for the legitimate transmission link. However, most of the AN-based PLS schemes rely on the the deployment of multiple antennas at the transmitter [28,29], which violates the low-cost and small-size requirements of IoT devices. To address this issue, the cooperative AN injection becomes a promising solution to ensure the IoT transmission security. Ref. [30] studied secure downlink transmission from a controller to an actuator with the help of a cooperative jammer to fight against multiple passive and non-colluding eavesdroppers. In [31], a wireless powered jammer is employed to improve the secrecy rate of an orthogonal frequency division multiplexing (OFDM) system, where the jammer operates with the harvest-then-jam protocol. In [32], a power allocation policy was developed to maximize the secrecy information rate while maintaining the harvested energy requirement of the energy receiver. The above works [30,31,32] are concerned about the design of the cooperative AN injection strategies. To reveal the security performance achieved by these strategies, Ref. [33] analyzed the secrecy outage probability of the system with multiple cooperative jammers and multiple eavesdroppers. The combination of AN injection with other secure transmission techniques can further improve the system security performance. Ref. [34] integrated the AN injection into the fountain coding based secure transmission framework. By transmitting the AN and the useful signal over two orthogonal channels, the intercept probability can be significantly lowered down and the legitimate user’s delay performance is guaranteed as well. Ref. [35] combined the compressive sensing technique with the AN injection approach, thus improving the system security performance while reducing the feedback overhead.

#### 2.2.2. Compressive Sensing

Compressive sensing (CS) can compress sparse signals with a much lower rate compared with the Nyquist sampling rate. Recently, the CS technique is utilized to realize physical layer security [26]. In CS, a linear transformation is applied to the sparse information-bearing signal by multiplying it with a measurement matrix. Transmission secrecy can be guaranteed if the measurement matrix is unknown to the eavesdropper. To achieve this goal, Ref. [36] proposed a scheme that utilizes an m sequence to construct the measurement matrix. The random seed used to generate the m sequence is distilled from the RSSI values of packets exchanged between the legitimate user pairs. Because the channel coefficient of the legitimate link is uncorrelated with that of the eavesdropping link, the eavesdropper cannot compute the same measurement matrix as the legitimate nodes and thus information secrecy can be guaranteed. In [37], a CS-based encryption scheme was developed for multi-carrier systems. In order to lower the probability of correct recovery of the measurement matrix by the adversary, the authors proposed transmitting artificial noise together with a sparse message. Furthermore, the channel state information (CSI) was exploited to selectively transmit an artificial noise such that its detrimental effect upon the legitimate receiver can be minimized. In [38], the security performance of the CS-based cryptosystem was studied. The analysis therein shows that the CS-based cryptosystem with circulant matrices over wireless channels can be computationally secure in terms of the indistinguishability, as long as the channel gains and the plaintext-to-noise ratio of the adversary are kept to be low for a long keystream and a short ciphertext. CS technique is also used to achieve physical layer security in multi-node cooperative systems, where the channel matrix between multiple sources and multiple relays is considered as the CS measurement matrix [39]. As is shown by [40], by adopting this method, the eavesdropper’s probability of signal recovery is zero. It should be noted that [39,40] only focused on the security transmission in dual-hop systems with CS. Different from these papers, Ref. [41] utilized the multi-hops to implement the projection process of CS, thus achieving keyless secure communication for multi-hop networks.

#### 2.2.3. Bit Flipping

The bit flipping technique is mainly applicable to securing the communications between the massive sensor nodes and the legitimate fusion center (LFC). In this approach, sensor nodes are divided into two groups (a strong group and a weak group) based on the strength of their channel gains to the LFC. The sensors with worse channel qualities, which are categorized into the weak group, are required to send the bit-flipped data, i.e., false data, to interfere with the eavesdropping fusion center (EFC), while the sensors with better channel qualities (i.e., those belonging to the strong group) are utilized to send the information-bearing data. Due to the statistical independence between the legitimate channel and the wiretap channel, with high probability, the received SNR at the EFC is much lower than that at the LFC, thus yielding a significant performance degradation at the EFC. Ref. [42] proposed a thresholding based bit flipping scheme. In this scheme, LFC first broadcasts two thresholds $\tau_{s}$ and $\tau_{w}$ to the sensors, which then compare their channel gains with the thresholds $\tau_{s}$ and $\tau_{w}$ to autonomously classify themselves into strong or weak groups. During actual data transmissions, the sensors that belong to the strong group (i.e., those satisfying $|h_{i}{|}^{2}>\tau_{s}$) transmit the real data, while the sensors that belong to the weak group (i.e., those satisfying $|h_{i}{|}^{2}<\tau_{w}$) send the bit-flipped false data to confuse the EFC. Following the work in [42,43], a three-threshold transmission scheme was further proposed where all the sensors are divided into three groups. Besides the sensors transmitting the real data and the false data, there are also some other sensors that remain silent during the transmission procedure. Compared with the scheme in [42], the three-threshold method in [43] further improves the energy utilization efficiency under the total energy constraint.

#### 2.2.4. Cooperative Secrecy

IoT is typically composed of massive physical objects such as sensors, controllers, and actuators. Although the processing capability of any single device is limited, the users’ secrecy requirements can still be satisfied by harnessing the cooperation among these low-power devices. The key idea of cooperative secrecy is to let the friendly nodes serve as jammers to send artificial interference to degrade the signal reception at the eavesdropper. Ref. [44] developed cooperative jamming (CJ) strategies for amplify-and-forward (AF) and decode-and-forward (DF) systems, respectively, where the relay nodes independently transmit the weighted artificial noises to worsen the eavesdropper channel. Ref. [30] combined secure beamforming with cooperative jamming to enhance the physical layer security. In this work, an optimization problem was formulated to minimize the secrecy outage probability subject to the secrecy rate requirement.

In addition to cooperative jamming, another popular cooperative secrecy approach is the secure relay selection technique. Ref. [45] developed a relay selection policy to select both the information forwarding helper and the friendly jammer, and introduced an adaptive mechanism to select the cooperation mode such that the secrecy outage probability can be minimized. Ref. [46] proposed a low-complexity single-relay selection scheme, taking both the transmission reliability and security into consideration. In [47,48], security-enhancing relay selection strategies were devised, where the impact of co-channel interference and the outdated CSI were investigated, respectively. Common to the works [45,46,47,48] is that all of them assume that the eavesdropper is an external malicious node in addition to the legitimate parties. Different from these papers, Refs. [49,50] considered secure transmission in dual-hop relaying networks where the intermediate relay nodes are untrustworthy. It has been proven that a positive secrecy rate can be achieved for this system by enlisting the help from the destination who performs cooperative jamming. Inspired by the pioneering works [49,50], plenty of papers have appeared that are dedicated to the PLS protocol design and performance analysis for untrusted relaying systems. In [51], the relay selection policy that maximizes the achievable secrecy rate was developed, and the scaling law of the network secrecy capacity was derived as well. In [52], the work of [51] was extended to a more realistic scenario where the information leakage during both the first phase and the second phase are considered. In [53], several relay selection policies were developed for successive AF relaying networks with untrusted nodes, where different complexity requirements were considered. The schemes described above are mainly applicable to the uplink transmission of IoT, where the sensor nodes send data to the central controller. For downlink transmission, Ref. [54] developed a cooperative privacy preserving method to prevent information leakage across the users. Through CSI-based AN design and cooperative AN injection, the data confidentiality of the desired user can be protected. To motivate the users to participate in cooperation, a user-grouping based selection criterion was also devised. Very recently, Ref. [55] proposed a constellation overlapping scheme to secure two-way untrusted relaying systems, where a truncation-channel-inversion based technique was introduced to make the signals from different terminal users experience the same equivalent channel, thereby realizing full constellation overlapping at the relay. Consequently, a high error floor is created, and data confidentiality is protected.

#### 2.2.5. Physical Layer Encryption

Instead of communicating a secret message straight away using the aforementioned PLS techniques, Alice and Bob can also opt to exploit the noisy channel to generate a secret key and use the key as a one-time pad to ensure information-theoretic security. From a practical perspective, the design of physical-layer encryption schemes from correlated channel observations turns out to be a simpler problem than the construction of codes for the wiretap channel. The procedure of physical layer key generation mainly includes four steps: (1) Channel probing: the communicating users measure the channel using a public pilot; (2) Parameter quantization: the analog to digital (binary) conversion; (3) Information reconciliation: error correction via public discussion; and (4) Privacy amplification: removing information leakage [56]. Ref. [57] presented a comprehensive review of the physical layer encryption techniques, highlighting the major technical challenges and solutions. Ref. [58] investigated the secret key generation issue for the ultra-wide band (UWB) channels, where the pulse response of the legitimate channel is used as the random source to distill the keys. Ref. [59] developed a key agreement strategy based on LLR thresholding. Ref. [60] utilized the received signal strength as the common randomness for the legitimate users, which enjoys a low implementation complexity. In addition to the above “deterministic” encryption techniques where the one-to-one mapping is required between the plain text and the cipher text, there is also another encryption method called probabilistic ciphering. As was described in [61], the sensor observations are randomly mapped to a set of discrete quantization levels, with the corresponding mapping probabilities only known to the LFC but unknown the EFC. By optimizing the probability distribution, a high error floor is created for the signal detection at the EFC, thereby guaranteeing transmission security. Ref. [62] applied the idea of probabilistic ciphering to distributed estimation, which significantly improves the system security performance.

### 2.3. Pros-and-Cons Analysis of the Existing PLS Techniques

Each of the aforementioned PLS techniques has its advantages and disadvantages. AN injection is easy to be implemented because the artificial noises can be produced using a pseudo-random number generator, for which many existing algorithms can be directly utilized. However, the secrecy gain offered by the AN injection approach is at the cost of additional energy consumption, which is used to send the artificial noise signal. Compared with the AN injection technique, the CS-based secure transmission method does not rely on the expenditure of additional power and is thus more energy efficient. However, a measurement matrix has to be shared between the legitimate transceivers while being kept secret from the eavesdroppers, which requires agreed knowledge about the CSI at different parties. This causes non-negligible overheads in the protocol design. The bit flipping technique can significantly reduce the implementation complexity and overcome the shortcomings of the CS-based approach. However, in the bit flipping method, the sensors within the weak group have to transmit the false data to confuse the eavesdropper, yielding a waste of power and bandwidth. Cooperative secrecy might be the most widely adopted PLS approach. The introduction of the cooperation mechanism offers the low-power devices the capability of combating the powerful eavesdroppers with less resource consumption. Nevertheless, the major disadvantage of the cooperative secrecy technique is that additional signaling is needed to coordinate different devices within the network, which complicates the protocol design as well. The physical layer encryption is essentially a cross-layer approach, which combines the secret key generation at the physical layer and the encryption at the application layer. The greatest asset of this approach is that it can be easily incorporated with the existing network security protocols, which is based on cryptographic techniques at the application layer. On the other hand, however, the effectiveness of the physical layer encryption heavily depends upon the fact that the communicating parties can reach an agreement on the generated keys, which is rather challenging in wireless environments.

## 3. Challenges of the PLS Protocol Design for Internet of Things

As mentioned previously, the research of PLS techniques has formed a large body of literature, ranging from fundamental information-theoretic studies to practical PLS protocol design. However, how to design PLS strategies that match the unique features of IoT well is still an open problem. The majority of the existing PLS techniques have the following drawbacks that prohibit their direct applications to IoT.

First, IoT devices are featured by “low-cost”, which means that these devices usually have very limited storage memory and processing capabilities. Moreover, the IoT devices are generally powered with batteries, which imposes significant energy constraints. The low cost and low power consumption features require that the PLS strategies must be highly energy-efficient, and can be implemented with very low complexity. However, the majority of the existing PLS schemes realize security with the cost of additional energy consumption or increased hardware complexity. For example, the AN based PLS approaches rely on the injection of artificial noise signals, which yields additional power consumption; the secure beamforming/precoding methods are typically based on the multi-antenna structure at the transmitter, which is infeasible considering the size and cost of IoT devices; the cooperation based PLS schemes require the existence of friendly jammers that send jamming signals, which also complicates the protocol design and increases the power consumption. From a practical point of view, the IoT-oriented PLS protocols have to take into account the resource constraints at the IoT devices, and make a trade-off among security, complexity, and energy consumption as well.

Second, from the networking perspective, IoT is expected to support wide-range transmission. For instance, in wireless sensor networks which is an embodiment of IoT, the local sensed data collected by sensors has to be delivered to the remote control center for further processing. However, the single-hop transmission distance in IoT is rather limited due to the low power of IoT devices. Therefore, to address the wide-range coverage requirement, the network transmission protocols have to incorporate many new features such as multi-hop routing, cooperative relaying, cognitive transmission, etc. This in turn requires the PLS strategies to be “smart” enough to adapt to complicated network environments. For example, the IoT devices have to cooperate with many untrusted immediate nodes. Although these untrusted nodes may not be malicious entities, it is possible that they are non-authenticated and with lower security clearance than the IoT devices. How can we exploit the untrusted nodes to assist information delivery while keeping the data content confidential to them? How does the number of untrusted relays impact the network security performance? These issues are not fully understood yet and more in-depth research is needed.

Third, IoT is targeted at providing massive connection capability which can accommodate millions of devices per square kilometer to exchange information. Moreover, the IoT devices, produced by different corporations, are heterogeneous nodes with totally different service types, traffic patterns, and transmission modes. These features make IoT a large-scale heterogenous network, for which the scalability issue is a primary concern. However, the existing PLS schemes are mainly developed for small-scale networks, for which only the link-level performance metrics are cared about, e.g., secrecy rate, information intercept probability, etc. Several fundamental problems for secure transmission in large-scale networks are not handled well. For example, how does the system secrecy rate scale with the number of nodes in the network? How can we translate the massive connection capability of the network into a powerful anti-eavesdropping resource? To answer these questions, new mathematical tools need to be developed, and innovative networking transmission protocols should be devised.

Fourth, future IoT is expected to support various application scenarios with diversified wireless services. Different types of services have totally different requirements on security, delay, throughput, and transmission reliability. However, the majority of the existing PLS protocols simply aim at optimizing the secrecy rate or the secrecy outage probability. Thus, it is impossible to provide comprehensive QoS assurance for IoT applications. To overcome this difficulty, the PLS protocol design should jointly consider various aspects of the user demands including delay, reliability, throughput, and secrecy as well.

## 4. Promising PLS Solutions for IoT

As described in the previous section, IoT has four unique features compared to traditional wireless networks: low cost, wide-range coverage, massive connection, and diversified services. In what follows, we would like to highlight three emerging PLS techniques that match these features well and have great potential in future applications.

### 4.1. Noise Aggregation and Self-Encryption

The key idea of physical layer security is to exploit the randomness of wireless channels to degrade the received signal quality at the eavesdropper. One popular method to achieve this target is to inject the AN into the null space of the legitimate channel. However, the use of AN results in an additional power consumption, which is not acceptable for battery-powered IoT devices. To address this issue, we proposed a novel method termed as noise aggregation [63], which works as follows: prior to data transmissions, each packet to be sent is assigned a positive number corresponding to the packet index. The odd-numbered packets and the even-numbered packets are transmitted within the odd and even slots, respectively. During data transmissions, the odd-numbered packets are directly sent out, and the even-numbered data packets are XORed with odd-numbered data packets that have been successfully decoded by the legitimate receiver. Due to the independence between the legitimate link and the eavesdropping link, the decoding of the odd-numbered data packets at the eavesdropper might be incorrect. As a result, the “decoding noise” from the odd slots will propagate to the even slots, and aggregate with the channel noise to worsen the detection performance of even-numbered packets at the eavesdropper. This is the so-called noise aggregation effect, which utilizes the natural noises rather than artificial noises to realize transmission security.

**Figure 1:**
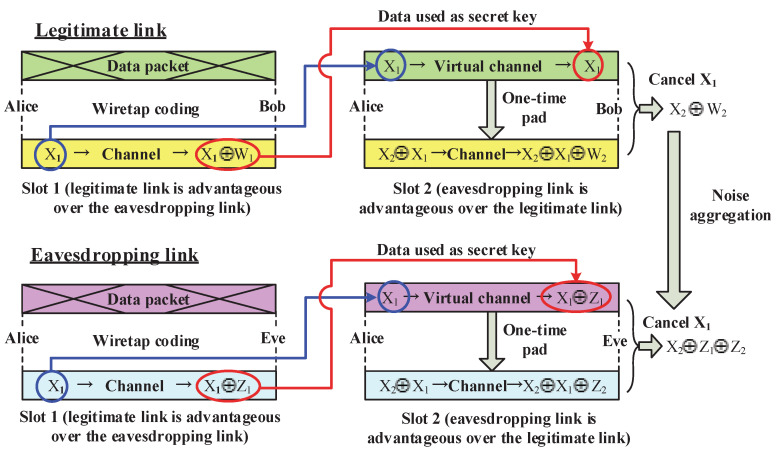
Illustration of the noise aggregation method.

The principle of the noise aggregation method can be illustrated in more detail by using Figure 1. For the ease of exposition, we assume that the transmissions from Alice to Bob are slotted, and each slot is of the same length. The wireless channel of the Alice–Bob link as well as that of the Alice–Eve link is modeled as a binary symmetric channel (BSC). It is further supposed that the channel fading state remains unchanged within any slot, and varies independently from slot to slot.

As illustrated by Figure 1, we consider two consecutive transmission slots. Without loss of generality, it is supposed that the channel quality of the legitimate link (Alice–Bob) is better than that of the eavesdropping link (Alice–Eve) within slot 1, while in slot 2, the channel quality of the legitimate link (Alice–Bob) is worse than that of the eavesdropping link (Alice–Eve). The detailed transmission procedure is described as follows.

(a) Within the first slot, the legitimate channel is advantageous over the eavesdropping channel. Hence, there is no need to perform the one time pad encryption. Instead, transmission secrecy of the source message is guaranteed by using the wiretap coding developed by information theoreticians. Assume the codeword transmitted from Alice is $X_{1}$. Then, the received codewords at Bob and Eve will be $X_{1}⊕W_{1}$ (the upper left part of Figure 1) and $X_{1}⊕Z_{1}$ (the lower left part of Figure 1), respectively, where $W_{1}$ and $Z_{1}$ are the corresponding channel noises, respectively.
(b) By exploiting the channel feedback from Bob to Alice, Alice can construct the capacity-achieving code to ensure that $X_{1}$ can be successfully decoded by Bob. Note that the eavesdropping channel is degraded compared to the legitimate channel during the 1st slot. Therefore, the decoding of $X_{1}$ at Eve is in failure. This motivates us that we can utilize $X_{1}$ as the key to encrypt the transmitted signal in slot 2. Assume the source data to be transmitted within slot 2 is $X_{2}$. Then, the encrypted data is $X_{2}⊕X_{1}$. At the end of this slot, the received codewords at Bob and Eve are expressed by $X_{2}⊕X_{1}⊕W_{2}$ (the upper right part of Figure 1) and $X_{2}⊕X_{1}⊕Z_{2}$ (the lower right part of Figure 1), respectively.
(c) Upon the reception of the signal $X_{2}⊕X_{1}⊕W_{2}$, Bob performs channel decoding to recover $X_{2}$. Since the “secret key” $X_{1}$ has already been obtained during slot 1, Bob can XOR $X_{1}$ with $X_{2}⊕X_{1}⊕W_{2}$ to produce $X_{2}⊕W_{2}$, which is the sufficient statics for the detection of $X_{2}$. Then, the signal detection at Bob is only impaired by the noise in slot 2. In contrast, the signals received at Eve during slot 1 and slot 2 are $X_{1}⊕Z_{1}$ and $X_{2}⊕X_{1}⊕Z_{2}$, respectively. After the XOR operation which cancels out the interference term $X_{1}$, the derived sufficient statics to detect $X_{2}$ will be $X_{2}⊕Z_{1}⊕Z_{2}$. Note that because $X_{1}$ and $X_{2}$ are independent variables that are both unknown to Eve, the observation $X_{1}⊕Z_{1}$ is statistically independent from $X_{2}⊕Z_{1}⊕Z_{2}$, which offers no information for the recovery of $X_{2}$. Obviously, the noises within both slot 1 and slot 2 are aggregated at Eve, thus degrading the signal detection performance of the eavesdropper.

Compared to the AN based approaches, the noise aggregation method does not depend on the use of artificial noise signals. Therefore, it is more energy-efficient and is thus appealing to IoT applications. On the other hand, in contrast with the traditional encryption method which relies on the exchange of dedicated secret keys, the noise aggregation approach is essentially a self-encryption technique which utilizes the previously transmitted messages to encrypt new messages. To realize the self-encryption, the transmitter only needs to perform the XOR operation, which is also advantageous in terms of implementation complexity. The noise aggregation based self-encryption approach has already been applied in several application scenarios. For instance, in [63], we developed a security enhancement scheme for video transmission, where the adoption of noise aggregation brings in approximately 1 dB SNR gain for Bob compared to Eve at the same level of frame error rate. In [64], we applied the noise aggregation technique in two-way untrusted relaying systems to realize a message-prioritization based unequal secrecy protection. Interested readers are suggested to refer to these two papers [63,64] for more information.

### 4.2. Anti-Eavesdropping Signal Design via Constellation Rotation

Constellation rotation was originally proposed in [65] as a diversity approach over fading channels. It has been also utilized as a powerful solution in interference cancellation [66,67] and cooperative spectrum sharing [68,69]. In [70], we developed a constellation-rotation based anti-eavesdropping method to secure the two-way untrusted relaying systems, where two users exchange information bidirectionally with an untrusted relay node. The key issue in designing PLS schemes for this system is to ensure no information leakage to the untrusted relay while still enlisting the relay’s ability in facilitating the two-way information delivery.

**Figure 2:**
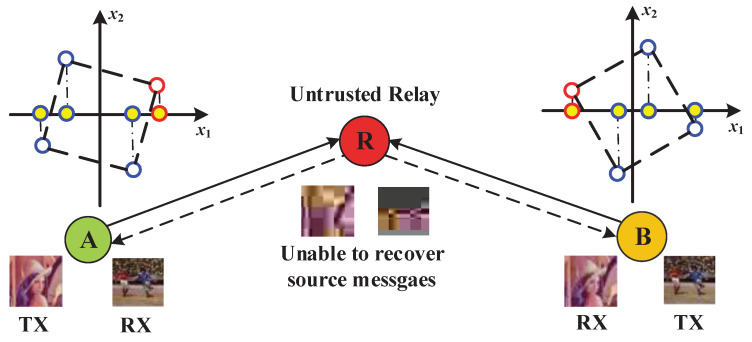
Two-way untrusted relaying system and the constellation rotation based secure transmission method.

Our key idea is to rotate the signal constellations. As is exhibited in Figure 2, the constellations employed at both terminal users are first rotated such that there exists a one-to-one mapping between the rotated signal constellation and its real or imaginary component. Then, only one dimension of the complex-valued signal is used to carry the user’s information, and the other dimension is used to send the artificial noise. By careful design as shown below, the AN from one user aligns with the information-bearing signal from the other user at the untrusted relay, which decreases the detection performance of the relay dramatically and keeps the users’ data confidential from the relay. From the terminal users’ perspective, the desired signal and the AN lie in different directions at either terminal user, and thus the signal detection can be realized without being affected by the interference. The constellation-rotation based secure transmission scheme is elaborated on in more detail as follows:

Let $x_{0}$ be the original constellation point taken from an alphabet X. Then, after constellation rotation, the resulting constellation point is $x=e^{j\theta }x_{0}$, with θ being the rotation angle. The choice of θ satisfies the condition that for any i≠k,

(3) $$ℜ\left\{x^{i}\right\}\neq ℜ\left\{x^{k}\right\},\;\;ℑ\left\{x^{i}\right\}\neq ℑ\left\{x^{k}\right\},\;\forall x^{i},x^{k}\in e^{j\theta }X.$$

That is, both the real and imaginary parts of any two symbols in the rotated constellation are different. In our proposed scheme, constellation rotation is first applied to every original symbol prior to data transmission.

As is shown from Figure 2, every cooperation period between user A and user B is composed of two phases. During the 1st phase, user A as well as user B first selects the information-bearing symbol from the rotated constellation set, and introduces the artificial noise to constitute the composite signal. Then, the composite signal is pre-coded with a coefficient to compensate the phase distortion in the transmission procedure. To be specific, the transmitted signals from user A and user B are expressed by

(4) $$S_{A}=\sqrt{\frac{P}{2}}\left(ℜ\left\{x_{A}\right\}+jw_{A}\right)e^{-j∠h_{\mathrm{AR}}}$$

and

(5) $$S_{B}=\sqrt{\frac{P}{2}}\left(w_{B}+jℜ\left\{x_{B}\right\}\right)e^{-j∠h_{\mathrm{BR}}},$$

respectively. In Labels (4) and (5), $x_{A}$ and $w_{A}$ ($x_{B}$ and $w_{B}$) represent the information-bearing symbol and the AN from user A (B), respectively, and $h_{\mathrm{AR}}$ ($h_{\mathrm{BR}}$) is the channel coefficient from user A (B) to relay R. The received signal at the relay node is expressed as

(6) $$\begin{array}{ccc} y_{R} & = & h_{\mathrm{AR}}S_{A}+h_{\mathrm{BR}}S_{B}+n_{R}\\ & = & \sqrt{\frac{P}{2}}\left(|h_{\mathrm{AR}}|ℜ\left\{x_{A}\right\}+\left|h_{\mathrm{BR}}\right|w_{B}\right)+j\sqrt{\frac{P}{2}}\left(|h_{\mathrm{BR}}|ℜ\left\{x_{B}\right\}+\left|h_{\mathrm{AR}}\right|w_{A}\right)+n_{R}, \end{array}$$

where $n_{R}$ denotes the additive noise at R.

Upon the reception of $y_{R}$, the relay might attempt to decode the signals sent from user A and user B. In order to extract user A’s signal, the untrusted relay has to first extract the real component of $y_{R}$ and then perform the maximum likelihood (ML) detection to estimate $ℜ\{x_{A}\}$. Due to the adoption of the constellation rotation, there is a one-to-one mapping between $ℜ\{x_{A}\}$ and $x_{A}$. Therefore, $x_{A}$ can be directly derived from $ℜ\{x_{A}\}$. Similarly, to obtain user B’s signal, the untrusted relay R extracts the imaginary part of $y_{R}$ to estimate $ℜ\{x_{B}\}$ and then recover $x_{B}$. It can be proven that, due to the injection of the artificial noise, the symbol error probability (SEP) at the untrusted relay in decoding user A’s information will not decrease with the increase of SNR, which yields an error floor at the untrusted relay. Similar analysis also holds for the detection of user B’s message. This result indicates that the proposed constellation-rotation based scheme can effectively deteriorate the SINR at the untrusted relay, thus enhancing the transmission secrecy.

During the 2nd phase, the relay amplifies the received signal and broadcasts it to users A and B. Having received the broadcast signal, user A as well as user B performs the ML signal detection as follows: the user first applies matched filtering to the received signal, and then extracts the real or imaginary component to perform self-interference cancellation, finally applies the ML decision to the processed signal. It has been shown in [70] that the received SINR at either terminal user is a monotonously increasing function of SNR. In other words, the SEP for the detection at the terminal users sharply decreases as the SNR increases, thus guaranteeing the transmission reliability. Therefore, the proposed constellation-rotation based scheme does not heavily affect the detection performance of the legitimate users.

From the implementation perspective, the main advantages of the proposed scheme can be summarized from the following aspects. First, through the rotation of signal constellations, the degree of freedom of the signal space can be fully exploited to enhance the data confidentiality, thus avoiding the excessive consumption of the power in transmitting artificial noise. Second, the rotation angles only depend on the adopted modulation format, and real-time calculations are not required to find or update the valued of the rotation angles, which yields a low implementation complexity. Third, compared with the physical-layer encryption methods which are based on secret keys, the proposed scheme does not need CSI-dependent key extraction or sharing between the legitimate users, thereby reducing system overhead significantly.

### 4.3. Fountain-Coding Based Secure Transmission

The fundamental framework for fountain-coding based transmissions [71,72,73] is described as below. The source file to be transmitted is first divided into packets with equal length, termed as information packets. Then, the transmitter encodes the information packets to produce the coded packets, and persistently sprays the coded packets towards the receiver. Here, each coded packet is the bit-by-bit XOR of several distinct source packets. Upon the reception of a coded packet, the receiver tries to decode by using some iterative decoding algorithms such as belief propagation [71,73]. As the iterative decoding proceeds, more and more information packets are recovered. Once the entire file is reconstructed, the receiver will send a feedback signal to inform the transmitter to stop producing new coded packets. Some well known fountain codes proposed so far include LT code [71], Raptor code [74], and Reed-Solomon (RS) code [75].

The essential point of exploiting fountain codes to enhance security is to expedite the decoding process at the legitimate receiver, such that the wiretapper cannot accumulate enough packets for the reconstruction of the entire source file. To be more specific, we assume the number of information packets that constitute the source data block to be *K*. Once the receiver has successfully received at least *K* independent coded packets, the entire file can be recovered [76]. This characteristic of fountain codes implies that, in wiretap channels, the data delivery from source to destination is secured if the destination can accumulate the *K* independent coded packets before the eavesdropper does. To achieve this goal, the unique features of the legitimate transmission, such as the error pattern, the CSI, and the data content characteristics, should be fully taken advantage of. Meanwhile, we should also assure that, this information, even known by the eavesdropper, cannot offer her any benefit.

The first work exploiting fountain codes for wireless security can be found in [77], where a truncated-channel-inversion based power control policy was developed to ensure a constant SNR at the legitimate receiver and a randomly varying SNR at the eavesdropper. In this manner, with high probability, the legitimate receiver can accumulate enough coded packets before the eavesdropper does, thus realizing transmission secrecy. The intercept probability of the fountain-coding aided secure transmission scheme was analyzed in [78], where an optimization model was also developed to minimize the intercept probability under delay and reliability constraints. Following similar ideas as [77], Ref. [79] applied random linear network coding (RLNC) [80,81] to realize secure layered video delivery [82], and devised a framework to prevent the eavesdropper from intercepting the enhancement layer video data. While [77,78,79] concentrated on a simple wiretap channel model with only three nodes, there are also several works studying fountain-coded secure transmission in cooperative relaying networks. In [34], we combined fountain coding and cooperative jamming to prevent information leakage in dual-hop decode-and-forward (DF) relay systems, where the constellation rotation technique was exploited to greatly deteriorate the received signal quality at the eavesdropper. Very recently, Ref. [83] extended the work of [34] to multi-relay networks, where relay-jammer selection was utilized to harvest the opportunistic relaying gain. Common to the existing works [77,78,79,80,81,82,83] is that all of them focus on how to exploit channel fading and physical-layer techniques to achieve a higher reception rate at the legitimate receiver. However, none of these papers investigates how to construct fountain code from the secrecy perspective. Differently from these works, Ref. [84] proposed a feedback-based mechanism to dynamically adjust the fountain encoder such that the decoding rate at the legitimate receiver can be improved. In our recent work [85], we developed a fountain-coding aided transmission scheme exploiting outage prediction and limited feedback, which has a very low implementation complexity and is thus applicable to future IoT. In what follows, we would like to present a brief introduction of this scheme.

**Figure 3:**
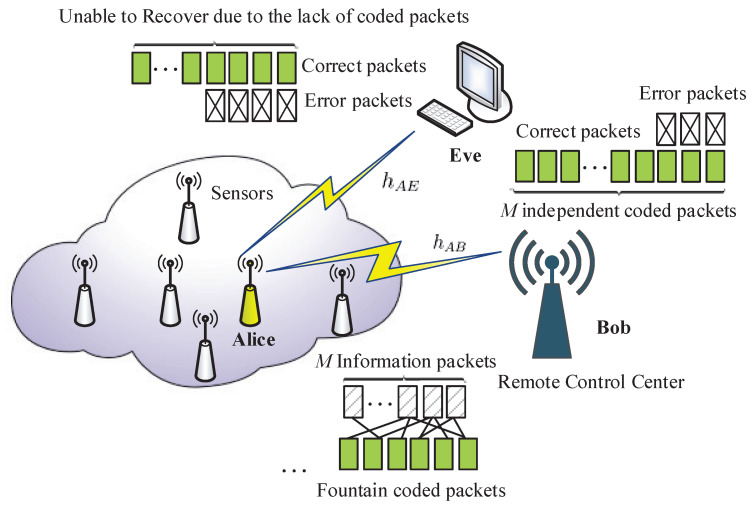
Fountain coding based secure transmission system.

As illustrated in Figure 3, we consider a wireless sensor network consisting of many sensor nodes, a central control unit Bob, and a passive eavesdropper Eve. The sensor node Alice wants to securely deliver a confidential file (the sensed data) to Bob. To achieve this goal, Alice first splits its file into *K* source packets denoted by $\left(u_{1},u_{2},\ldots ,u_{K}\right)$. Then, fountain coding is employed to encode these packets into a potentially infinite number of fountain packets $\left(v_{1},v_{2},\ldots \right)$. Finally, these fountain packets are further encoded by capacity-achieving code at the physical layer to produce the transmitted packets $\left(p_{1},p_{2},\ldots \right)$. To better illustrate our idea, we focus on two consecutive transmission slots, say slot t−1 and slot *t*. Within time-slot t−1, upon the reception of the transmitted packet $p_{t-1}$, Bob predicts the conditional outage probability (COP) of slot *t* based on the current channel state and the specific channel model. If the predicted COP is lower than a predetermined outage threshold, the fountain coding strategy in slot *t* will be given by

(7) $$v_{t}=u_{d,1}⊕u_{d,2}⊕\ldots ⊕u_{d,k}⊕u_{n,p},$$

where $u_{d,1},\ldots ,u_{d,k}$ represent all the source packets that have been recovered by Bob until slot t−1, and $u_{n,p}$ is a randomly-chosen source packet that has not been recovered by Bob yet. The rationale behind this policy is as follows. If the predicted COP is lower than the threshold, a good channel quality is expected for the legitimate link in the next slot. The coding strategy in Label (7) ensures the instant recovery of a new source packet once the next-slot transmission is successful. On the contrary, if the predicted COP is higher than the threshold, implying that the transmission over the legitimate link will be in failure with high probability, the fountain coding strategy in slot *t* should be

(8) $$v_{t}=u_{1}⊕u_{2}⊕\ldots ⊕u_{K},$$

which is the XOR of all the source packets. In this manner, the eavesdropper is prevented from extracting a new source packet from its received signal.

With the proposed strategy, the fountain encoder matches well with the time-varying channel conditions of the legitimate link such that the receiver can achieve a much higher decoding rate for the source packets compared to the eavesdropper, thus guaranteeing transmission secrecy. The complexity of the fountain coding based secure transmission scheme mainly includes two parts. First, fountain encoding/decoding is required at the application layer. Second, channel feedback is needed from Bob to Alice to dynamically adjust the fountain encoder structure. However, the fountain encoding/decoding can be realized by using several mature algorithms with linear time complexity. Besides that, only two bits are needed to be fed back. One bit is used to inform Alice about the outage prediction result, and the other bit is used to indicate the decoding status (success or failure) of the currently-received coded packet. Therefore, the additional overhead of the proposed scheme is trivial, which can be affordable for most IoT applications.

### 4.4. Summary of the IoT-Oriented PLS Solutions

Compared with the classical PLS strategies, the schemes described in Section 4 are better suited to the Internet of Things, the reasons of which are summarized as follows. First, the schemes developed in Section 4 are energy efficient, thus satisfying the low energy consumption requirements of IoT. To be specific, the noise aggregation approach utilizes the intrinsic noise in wireless channels, rather than the artificial noise, to degrade the eavesdropper’s received signal quality, and guarantees transmission secrecy without the cost of additional power. The constellation rotation method creates an irreducible error floor at the untrusted relay via the optimized design of signal constellation, thus avoiding the excessive consumption of energy in generating the artificial noise. The fountain-coding aided strategy realizes secrecy by exploiting the characteristics of fountain coded transmission that the entire file can be recovered only if a sufficient number of coded packets can be accumulated. This method does not require any additional energy to guarantee secrecy. Second, the three schemes described in Section 4 enjoy very low implementation complexity, which matches the low-cost feature of IoT devices well. As stated previously, noise aggregation is essentially a self-encryption technique that utilizes the previously transmitted messages to encrypt new messages. Only a simple XOR operation is required at the transmitter, which is affordable by the IoT devices. In the constellation rotation scheme, the constellation rotation operation incurs negligible overhead. Moreover, the rotation angles only depend on the adopted modulation formats and can be calculated offline prior to data transmissions, yielding trivial computational load as well. The overheads involved in the fountain coding based scheme mainly include the fountain encoding/decoding operations and channel feedback from Bob to Alice. However, the fountain encoding/decoding algorithm has a linear time complexity, and the amount of feedback within any slot is only two bits. Thus, the fountain-coding based scheme is also attractive for IoT applications. Third, from the perspective of IoT services, the schemes discussed in Section 4 are also more competitive compared to the classical PLS solutions. In particular, by using the noise aggregation approach, the message-prioritization based unequal secrecy protection can be realized, which is of practical significance to the video surveillance applications. In the constellation rotation scheme, the power ratio of the information-bearing signal to the artificial noise in the composite signals (4) and (5) can be adaptively adjusted, thereby providing a flexible trade-off between the reliability and security performances. Finally, in the fountain-coding based secure transmission scheme, the outage threshold can be optimized such that various aspects of the system QoS requirements can be balanced, including secrecy, throughput, as well as transmission delay.

Although the emerging PLS techniques described in Section 4 have notable advantages, there are also some disadvantages associated with them. Briefly speaking, all of the three strategies require channel feedback or information exchange between the legitimate entities, which causes a slight degradation of the rete performance of the legitimate system. Therefore, these schemes are not suitable to real-time applications where the data delivery has stringent delay requirements. However, IoT services are typically with very low data rates. Thus, the disadvantages of the schemes do not hamper their adoption in future IoT.

In Table 1, we summarize the advantages and disadvantages of all the PLS techniques discussed in Section 2 and Section 4, highlighting the energy consumption and implementation complexity aspects. We also show the potential application scenarios to which each candidate PLS solution applies. Table 1 can be used as a guideline that instructs us to select the PLS solutions for various IoT applications.

## 5. Conclusions and Future Directions

In this article, a comprehensive review of the physical layer security techniques in the Internet of Things was presented. The features as well as security requirements of IoT were first discussed. After that, the basic principle of physical layer security was introduced, and several representative IoT-oriented physical-layer-security technical solutions were summarized. Finally, we analyzed the challenges faced by IoT secure transmission protocol design, and introduced three emerging PLS solutions that can well address these challenges.

Although the research on physical layer security has generated a large body of literature, with the work ranging from fundamental information-theoretic analysis to practical PLS strategy design, it is still challenging to develop PLS schemes that satisfy the multi-dimensional requirements of future IoT. Some issues that are worthy of further studies are listed as follows:

(1) The PLS scheme design to combat active attacks. Until now, the majority of PLS solutions focus on the anti-eavesdropping techniques. However, eavesdropping is just a simple and passive attack form. In future IoT, there will be various forms of active malicious attacks, e.g., message modification, information disclosure, pilot spoofing, jamming, masquerade attack, etc. It remains an open problem as to how to exploit the PLS techniques to deal with these attacks. Cross-layer design may be a promising method to address this issue.
(2) The new metric for performance evaluation. In PLS research, the widely adopted performance metrics include the achievable secrecy rate and the secrecy outage probability. These metrics are proposed from an information-theoretical point of view. In future IoT, the heterogeneity of the devices and the services cause the diversity in user demands. This in turn calls for the proposal of new metric for evaluating the performance of PLS schemes. The new metric should take the multi-dimensional user requirements, e.g., secrecy, delay, throughput, packet loss rate, etc., into consideration, and give a comprehensive evaluation of the developed schemes.
(3) The use of PLS techniques to new systems and scenarios. Current studies are mainly concerned about the wireless sensor networks as an application scenario. In future IoT, several new systems are emerging, and many new application scenarios will be considered, e.g., the backscatter systems. Thus, innovative research works are needed, including new models, new analytical tools, etc.

## Figures and Tables

**Table 1:** Comparison of the Physical Layer Security Techniques.

PLS Technique	Advantages	Disadvantages	Implementation Complexity	Energy Consumption	Potential Application Scenarios
AN injection	AN generation can be easily realized	Additional energy consumption	Moderate	High	Telemedicine
Compressive sensing	No need for additional power	Measurement matrix has to be shared	High	Low	Wireless body area networks
Bit flipping	Signal processing operation at the transmitter is simple	Extra bandwidth and energy is needed	Low	High	Sensor networks
Cooperative secrecy	Highly flexible and better security performance	Significant signaling overhead	High	Moderate	Unmanned aerial vehicle communications (UAV)
Physical layer encryption	Easily incorporated with existing security protocols	Channel probing and secret key agreement is needed	High	Low	Remote coaching
Noise aggregation	Easy to be implemented	Channel feedback is needed	Low	Low	Immersive systems, video surveillance
Constellation rotation	Degree-of-freedom of the channels can be fully exploited	CSIT is needed	Moderate	Moderate	Device-to-device communications (D2D)
Fountain coding	Comprehensive QoS guarantee	Channel feedback is needed	Low	Low	Industrial Internet of Things
